# Morbid Anatomy.—Case of Death from an Encysted Tumor in the Brain

**Published:** 1823-12

**Authors:** F. Bush


Art. IX.—Morbid Anatomy.—*Case of Death from an encysted Tumor in the Brain.* Communicated by F. Bush, Esq.

Wm. Grist, aged eighteen years, by trade a tailor, having been much exposed to cold in a garret where he worked, complained of being unwell: shortly afterward he had a fit, which continued for some time. I did not see him in it, but, from the account I obtained, it was epileptic. He applied to me about four months ago, which was many weeks after the fit, complaining of slight head-ache, and a discharge of pus from the ear. From having been lively, he appeared depressed in spirit, and without energy to enable him to pursue his work. The pulse was natural as to strength and frequency. The complaint continued in appearance to be stationary for some weeks, till his vision became imperfect: the left eye was drawn from its natural position, and, to look at objects, he was obliged to shut one eye; but could see equally well with either when the other was shut, so that the focal distance appears to have been deranged.

There were evident proofs of some mischief going on in the head; and, notwithstanding the use of bleeding, blisters, setons, mercurials, purgatives, and diuretics, no abatement of disease took place: in truth, he gradually grew worse, although his appetite was good, and he was capable of using walking exercise at times. For the last month before his decease, he laboured under occasional opisthotonos: this symptom was not constant, but continued for some hours, and then went off. It may be worthy of remark, that it was usually accompanied by sickness. For some time before he died, he expressed himself as having great pain in the head, sometimes for an hour or two, at others for a longer period; but, even up to the time of his death, he was occasionally pretty well, and able to walk out, and the day before that event he walked, to see some relations, a distance of near a mile. In the night after, he was seized with opisthotonos, and expired in a few hours.

Permission being obtained to examine the head, it was done on the following morning. Nothing indicating disease occurred on removing a portion of the cranium, including parts of the frontal, two parietal, and occipetal bones; the dura mater was not marked by diseased appearance; the pia mater was also without traces of disease; and so was the substance of the brain, till on slicing it down to near the lateral ventricle, when a firm substance was found imbedded in the brain: further examination showed it to be a cyst, containing about two ounces of purulent matter; it was pyriform, and attached by a very small base to the pætrous ridge of the temporal bone.

*Remarks.*—It may be worthy of observation, that the pulse was natural, the tongue clean, the bowels regular, and the appetite tolerably good, during the whole progress of this formidable disease; and that it did not produce (except the tetanic affection, which existed occasionally for a short time, and then went off, and the strabismus, which was permanent,) either paralysis of any voluntary muscles, or derangement in the sanguiferous or respiratory organs.

This case illustrates to what extent the brain will accommodate itself to the gradual formation of an adventitious substance of great bulk, without manifesting any of those symptoms which are known to arise from the pressure occasioned by the sudden extravasation of even a very small quantity of blood on the brain.

### *A Case of Recovery from Intussusception, where fifteen or eighteen Inches of the Ilium separated, and was discharged per Anum.*

J. Coombs, a lad twelve years of age, was attacked with pain in the umbilical region: some mild laxative was given, without affording relief. On the second day I saw him: the disease was marked by sickness, vomiting, severe pain extending over the abdomen, a dry brown tongue, skin extremely hot, and the pulse corded, hard, and small, making 120 beats in the minute. The case was clearly marked as being inflammation of the bowels: blooding, laxatives, the warm bath, and blisters, with the usual remedies for the subjugating such a disease, were used without benefit. On the fourth day, when he appeared much exhausted, and his dissolution was expected, a considerable quantity of feculent discharge, mixed with blood, took place per anum: from this time the bowels were easily acted on by the mildest laxatives, the sickness left him, the pulse became less frequent, and his pain inconsiderable. Matters went on thus favourably until the eighth day from this attack, when a portion of bowel was voided with a common alvine discharge: this portion, fifteen or eighteen inches long, was part of the ilium. From this time his convalescence was regular and rapid; but he complained of pain in attempting to gain the erect position, and was obliged to keep himself bent forward for three or four weeks, at which time he had regained his health; the bowels being regular, but rather relaxed.

About twelve weeks after his complete recovery, he was attacked with typhous fever, of which he died a fortnight after the seizure. I availed myself of the opportunity to examine the contents of the abdomen after death. The traces of the diseased bowel were visible, by a considerable puckering and contraction where the slough had taken place, and the parts had united; yet there did appear sufficient room for the feces to pass, and, but for the febrile attack, he might have enjoyed ordinary health. The part that formed the intussusception, and which came off with the alvine discharge, together with part of the mesentery, and the alvine portion where the union had taken place, I thought worthy of some anatomical collection, and therefore took the liberty to send it to Sir A. Cooper.

